# Effect of Celastrus paniculatus on Alcohol-Elicited Conditioned Place Preference and Alcohol Withdrawal-Induced Anxiety in Mice

**DOI:** 10.7759/cureus.81962

**Published:** 2025-04-09

**Authors:** Sonali Satam, Sanket Raut, Yashashri C Shetty, Padmaja A Marathe, Alhad Mulkalwar, Ashwinikumar Raut, Nirmala Rege

**Affiliations:** 1 Department of Pharmacology and Therapeutics, King Edward Memorial Hospital and Seth Gordhandas Sunderdas Medical College, Mumbai, IND; 2 School of Psychology, University of Tasmania, Tasmania, AUS; 3 Department of Pharmacology, Dr. D.Y. Patil Medical College, Hospital and Research Centre, Dr. D.Y. Patil Vidyapeeth (Deemed to be University) Pimpri, Pune, IND; 4 Clinical Research and Integrative Medicine, Medical Research Centre of Kasturba Health Society, Mumbai, IND

**Keywords:** anxiety, dependence, diazepam, intermittent access, naltrexone

## Abstract

Background

*Celastrus paniculatus *(CP), an Indian medicinal plant, is hypothesized to be effective in alcohol use disorder (AUD) based on its medicinal properties useful in CNS disorders as per ancient Ayurveda literature. Hence, it was tested in animal models of AUD.

Methodology

The study was conducted in Swiss albino mice (n=144) of either sex after obtaining Institutional Animal Ethics Committee permission. Three doses of seed oil of CP (CP1: 140, CP2: 280 and CP3: 560 mg/kg) were administered orally in milk (vehicle for CP). A conditioned place preference (CPP) model was used to study the effect of CP on alcohol dependence. Elevated plus maze (EPM) and open field test (OFT) were used to study its effect on alcohol withdrawal-induced anxiety. Naltrexone and diazepam were used as positive controls.

Results

The results in all the CP groups (CP1 (337.88±20.66s, p<0.05); CP2 (322.38±17.61s, p<0.05) and CP3 (315.50±4.24s) as evaluated by the time spent in alcohol paired compartment in CPP) showed better protection against alcohol dependence as compared to the vehicle (552.63±27.47s, p<0.05). The values in all the CP groups were comparable to the naltrexone group. In the EPM test, the CP3 group showed significantly increased time spent (170.63±19.75s) in the open arm (*vs *the vehicle control, 12.75±11.03s, p<0.05). Also in OFT, longer time was spent in the central zone by mice in the CP3 group (23.25±6.19s *vs.*8.50±5.48s in the vehicle group, p<0.05). The results of the CP3 group were comparable to diazepam in EPM and OFT experiments.

Conclusion

*Celastrus paniculatus* seed oil showed a dose-dependent effect in the CPP model of alcohol dependence in mice. The highest dose of CP also prevented alcohol withdrawal-induced anxiety.

## Introduction

Alcohol drinking is common all across the world. It is reported that approximately two billion people consume alcohol and one-third of those who take alcohol as part of social drinking (approximately 76.3 million) are likely to develop alcohol use disorder (AUD) [1]. The Global Status Report of WHO published in 2014 stated that 7.5% of individuals in the age group of 15 years or more were suffering from AUD, with the highest prevalence being reported from the European countries [1]. The Indian National Mental Health Survey of 2016 reported that the prevalence of alcohol dependence was rising (4.6%) [2]. Naltrexone, disulfiram, topiramate and acamprosate are the drugs from modern medicine which are used in the treatment of AUD in addition to cognitive behavioral therapy (CBT) [3,4]. The existing therapy for AUD has limitations. One study has reported that naltrexone is associated with a high relapse rate of 40% [5]. In another ‘COMBINE’ study, acamprosate did not reduce alcohol consumption when compared to the placebo, alone and also when combined with CBT or with naltrexone [6].

We are far from having an ideal therapy for the treatment of AUD. Hence, there is a need to explore new treatment options for AUD [5-7]. Several Indian traditional medicines, such as *Withania somnifera* (L.) Dunal and *Silybum marianum*, as well as biologically active compounds isolated from these traditional medicines, have shown promising effects on alcohol consumption in animal studies [8,9].

The field of Indian medicinal plants has not been explored to a great extent to date for systematic search of drugs for the treatment of AUD. *Celastrus paniculatus* (CP), which is also known in Ayurveda as Jyotishmati or Malkangni, belongs to the family of Celastraceae. It is found in the tropical dry deciduous forests and hilly areas in India. CP is also native to the wild forests of countries like Nepal, Sri Lanka, Australia, China, Thailand and Malaysia [10]. Traditionally, its seeds are used in Ayurvedic practice for memory sharpening, cognitive improvement and enhancement of intellect [11,12]. As per Ayurveda, a number of other conditions such as skin ailments (ulcers, scabies, pruritis, wounds, leukoderma), headache, joint pains, dysmenorrhoea, and epilepsy are also treated using the seed and seed oil of CP [11].

It has been reported that an excess of glutamate in the brain reward pathway contributes to the development of alcohol dependence [13]. In a study by Godkar et al. the seed extract of CP inhibited glutamate-induced neurotoxicity in rats [14]. In another study reported from the literature, glutamate-induced neurotoxicity in the prefrontal cortex was shown to impair decision-making, which is seen in AUD [15]. In a study conducted by Nalini et al. to assess the effects of CP on rat-brain neurotransmitters, CP was shown to decrease the concentration of dopamine in the brain [16]. Since the final common mechanism through which different addictive substances cause dependence is through the release of dopamine in the nucleus accumbens [17], it is postulated that CP may have a role in the treatment of AUD by virtue of its action on glutamatergic and dopaminergic pathways. Hence, it was decided to explore the utility of Celastrus paniculatus in animal models of AUD.

## Materials and methods

The Institutional Animal Ethics Committee of Seth Gordhandas Sunderdas Medical College and King Edward Memorial Hospital, Mumbai (AEC/02/2014) had approved the study which was carried out in accordance with the Committee for the Purpose of Control and Supervision of Experiments on Animals (CPCSEA) guidelines. Swiss albino mice of either sex (n=144) weighing 20-30 gm were procured from the Institute’s Central Animal House. The mice were housed in cages as four mice/cage during the study period.

Study drugs

The test drug, CP seed oil, was procured from M/s. Pharmanza Herbal Private Limited, Anand, Gujarat and positive controls naltrexone and diazepam were procured from Sigma Aldrich, Bangalore, India (Extractive yield value: 0.25%; Certificate of Analysis no. SF/QC/CHI/026-04.02). The doses of CP administered to mice in the conditioned place preference (CPP) model were selected from the doses administered in rats reported in literature by Gattu M et al. [17]. The doses were 100 mg/kg, 200 mg/kg and 400 mg/kg for rats and the same were extrapolated to mice using the dose conversion formula given by Pagett and Barnes [18]. The three doses of CP were used to study the dose-dependent effect in mice and to choose the most effective dose for the second part of the study.

Study procedure

The study was divided into two parts. In the first part, a conditioned place preference model was used to evaluate the effect of CP on development of alcohol dependence. In part two, two models of anxiety: elevated plus maze test and open field test, were used to assess whether CP reduces alcohol withdrawal-induced anxiety.

Part 1: Conditioned place preference (CPP) model

Experimental Groups and Drug Administration

The mice were divided into six groups: Group 1 was administered distilled water (as a vehicle); Group 2: CP 1 (seed oil in milk, low dose of 140 mg/kg, orally); Group 3: CP 2 (seed oil in milk, medium dose 280 mg/kg, orally); Group 4: CP 3 (seed oil in milk, high dose 560 mg/kg, orally) [17]; Group 5: naltrexone (5 mg/kg, orally) [19]; Group 6: vehicle for CP (milk, 2 ml orally). 

CPP Model 

The CPP apparatus has two cuboidal compartments (30 cm × 15 cm × 20 cm). The compartments are made of an opaque plastic substance. The two compartments differ from one another as each compartment has unique visual and tactile cues. The cues provide conditioned stimuli. The experiment was conducted for 11 consecutive days. On day 1 (Adaptation day), mice were carried into the testing room, weighed and handled by the experimenter so as to reduce the novelty of the environment and stress associated with handling and injections. On day 2 (Pre-conditioning day), the mice were placed on the border of both compartments. They were allowed to explore both compartments for 900 seconds. Using an infrared beam sensor, time spent by a mouse in each compartment was recorded over a period of 15 minutes. Of the two compartments, the one in which the mouse spent more time was considered as preferred compartment for that mouse while the other one was the non-preferred compartment. The apparatus was cleaned every time before a mouse was introduced. This was done to ensure that there are no traces of urine and fecal matter. From days 3-10, the mice were conditioned using alcohol to stay in non-preferred compartment by alternately subjecting them to four alcohol-paired days (days 3, 5, 7 and 9) and four saline-paired days (days 4, 6, 8 and 10). The mice were placed in the alcohol-paired compartment or non-preferred compartment for 30 minutes immediately after intraperitoneal (i.p.) administration of alcohol (2 mg/kg) and placed in the saline-paired or preferred compartment after administering saline. Milk as vehicle, CP seed oil, and naltrexone were administered every day, 30 minutes before i.p. injection of alcohol (2 mg/kg) or saline. On the 11th day (day of final conditioning session), a post-conditioning test was conducted identical to the pre-conditioning test for 900 seconds (15 minutes). The difference in the time spent in the alcohol paired compartment during pre-conditioning and post-conditioning tests was noted for each animal from all the experimental study groups [20].

Part 2: Effect of CP on alcohol withdrawal-induced anxiety

Experimental Groups

The dose of CP showing the highest effect in part 1 was used in the second part of the study. The mice were divided into three groups: Group 1 was administered vehicle (milk, orally); Group 2: CP 3 (seed oil in milk, 560 mg/kg orally); Group 3: diazepam (1 mg/kg orally) [21].

Intermittent-Access 20% Ethanol 2-Bottle-Choice Drinking Paradigm 

Alcohol addiction was induced by intermittent access to 20% ethanol via a two-bottle choice drinking paradigm. Thrice a week, the mice were given access to ethanol during a 24-hour session. On Monday, following the end of the housing acclimatization period, each ethanol-naïve mouse was given access to one bottle of 20% v/v ethanol and one bottle of water. After 24 hours, the bottle containing ethanol was replaced with a second bottle of water which was made available for the next 24 hours. This pattern was repeated on Wednesdays and Fridays. The mice had unrestricted access to water on Tuesdays and Thursdays. The mice also had unlimited access to two bottles of water over the weekend after 24-hour measurements were taken on Saturday morning. All groups received alcohol for two weeks as described above. Test drugs were administered three days post-ethanol withdrawal. The antianxiety effect was studied on day 4 [22].

Elevated Plus Maze Test (EPM) 

The elevated plus maze consists of two open and two closed arms. The open pair lies perpendicular to the closed one. The maze is located 29 cm above a black floor. A mouse was placed at the centre of the plus maze and was allowed to explore it for a period of five minutes. Time (in seconds) spent by animals in the open and closed arms was recorded. The total number of open arm entries with percentage preference for open arm was also evaluated [23].

Open Field Test (OFT) 

The OFT test is carried out in a dark and soundproof room. During the experiment, a mouse was placed against one of the walls of the apparatus. The mouse was allowed to explore the apparatus for five minutes and then it was returned to its cage. The study drugs were administered 30 minutes (for intraperitoneal route of administration) or 60 minutes (for oral route of administration) prior to exposing the mice to the open field. The time spent in peripheral zone, central zone, number of entries in peripheral zone and central zone were calculated [24].

## Results

Part 1: Conditioned place preference model

**Figure 1:**
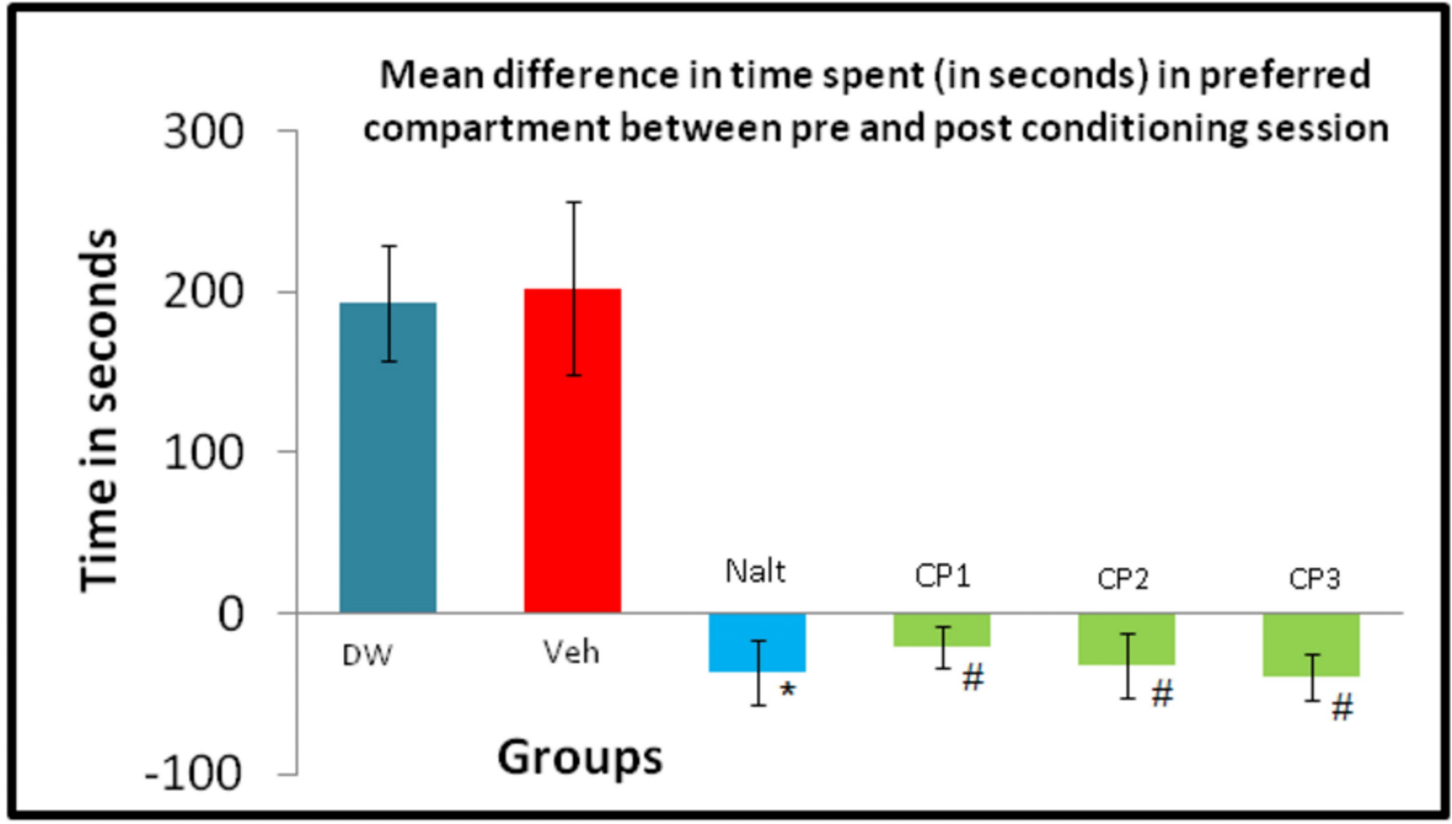
Mean time spent in pre- and post-conditioning session in conditioned place preference (CPP) *p< 0.05 vs. DW; using ANOVA and post hoc Tukey’s test, # p< 0.05 vehicle (milk) using ANOVA and post hoc Tukey’s test CP1 - Celastrus paniculatus group 1, CP2 - Celastrus paniculatus group 2, CP3-  Celastrus paniculatus group 3, Nalt - Naltrexone, DW - Distilled water

The data shown in Figure 1 is the mean difference in the time spent in the alcohol-paired compartment during pre- and post-conditioning sessions in all the groups. All three CP groups and the standard control naltrexone reduced the mean difference in time spent in the alcohol-preferred compartment as compared to their respective control which was statistically significant (P<0.05). It is observed that the highest dose of CP was most effective and there was a dose-dependent response observed within the groups. All the doses of CP showed comparable results to the naltrexone group.

Part 2: Withdrawal-induced anxiety model

Elevated Plus Maze Test 

**Figure 2:**
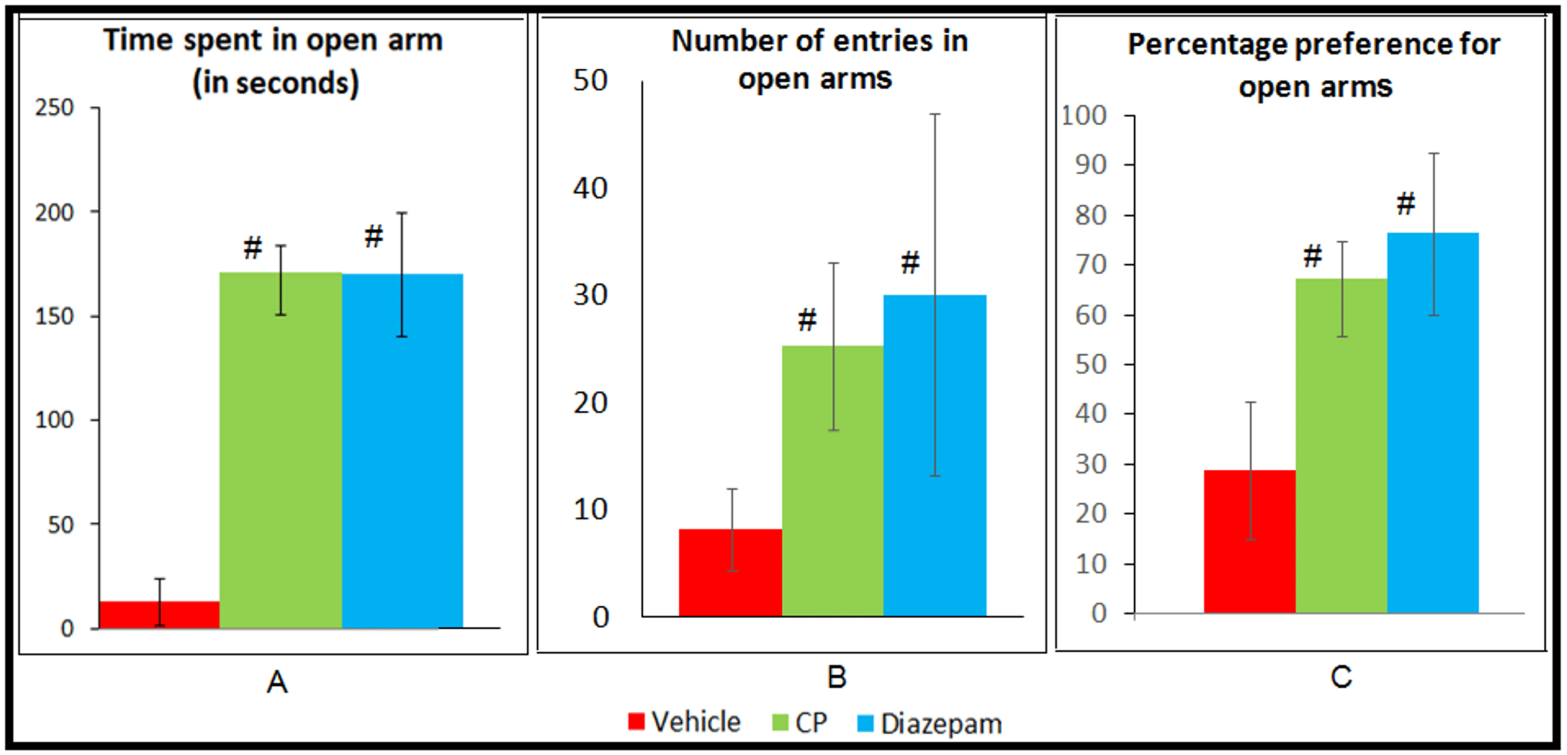
Effect of CP in EPM on a. Time spent in open arm, b. Number of entries in open arm and c. Percentage preference for open arm ^#^p<0.05 vs. Vehicle Group using ANOVA and post hoc Tukey’s test CP - Celastrus paniculatus, EPM - elevated plus maze, Vehicle - Vehicle for CP (Milk)

Analysis done by one way ANOVA and post hoc Tukey’s test showed that the Celastrus paniculatus group mice when compared to those of vehicle group spent significantly more time in open arms (170.63±19.75 s vs. 12.75±11.03 s, p<0.05), had more no. of entries in open arm (25.38±7.87 vs. 8.25±3.85, p<0.05) and had greater percentage preference for the open arms (67.156±11.54% vs. 28.58±13.81%, p<0.05). The results of Celastrus paniculatus group were comparable with the diazepam group (dose, 2 mg/kg) for all the variables (Figure 2).

Open Field Test 

**Figure 3:**
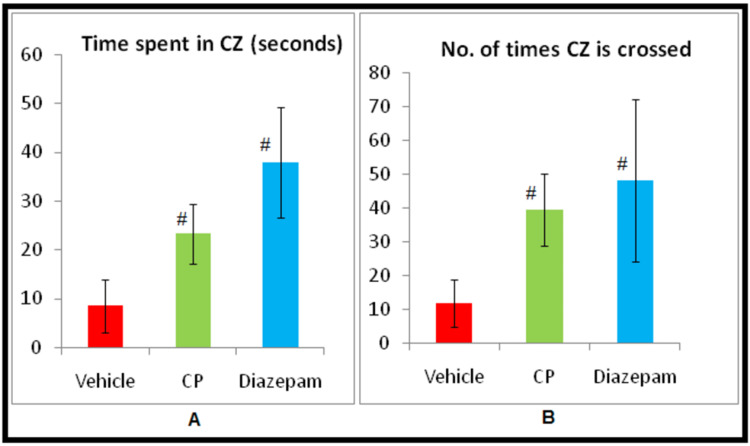
Effect of CP in OFT on a. Time spent in the CZ (Central zone) b. Number of times central zone (central square) is crossed ^#^p<0.05 vs. Vehicle Group using ANOVA and post hoc Tukey’s test CP - Celastrus paniculatus; OFT - open field test; Vehicle - Vehicle for CP (Milk)

Analysis done by one way ANOVA and post hoc Tukey’s test showed that the Celastrus paniculatus treated mice preferred central zone (23.25±6.19 s vs. 8.50±5.48 s, p<0.05), had greater frequency of crossing the central square (39.38±10.64 vs. 11.75±7.02, p<0.05) along with a significant fall in the number of stretch attend postures (10.00±3.89 vs. 25.25±8.41, p<0.05). The results of Celastrus paniculatus group were comparable with the diazepam group (dose, 2 mg/kg) for all the variables (Figure 3).

## Discussion

CP is classified as a medhya rasayana group of herbs (the word "Medhya" means intellect or retention, while "Rasayana" means therapeutic preparation), and it has been used by practitioners of Ayurveda for memory improvement [9]. The extensive review by Sankaramourthy et al. provides evidence regarding the promising role of CP against common neurological disorders [25]. However, the role of CP has not been studied in AUD so far. Hence the present study was conceived.

The development of alcohol addiction is a complex and dynamic process. Continued excessive alcohol consumption can lead to development of dependence that is associated with a withdrawal syndrome when alcohol consumption is ceased or substantially reduced [26]. The drive to drink alcohol is predominantly to seek the rewarding effects of alcohol for some individuals and to avoid the negative aspects of its withdrawal for others [27].

Since a deﬁnitive single animal model of ‘alcoholism’ does not exist, animal models that mimic particular aspects of behavioral spectrum which are fundamental to the addiction process in humans were used in the study [26]. We chose to study the effect of CP in an animal model of alcohol dependence using CPP and alcohol withdrawal-induced anxiety (EPM and OFT) as both dependence and withdrawal-induced anxiety contribute to relapse and sustained use of alcohol in humans. The CPP model helps to study the effect of the test drug against reinforcing property of alcohol EPM and OFT help to assess whether the test drug is able to reverse the negative affective aspects of alcohol withdrawal. In our study, all three doses of CP showed a positive effect in the CPP model. The effect of CP could be mediated by virtue of its action on decreasing the brain dopamine levels. Nalini et al. have demonstrated that administration of CP is associated with decreased brain dopamine levels which substantiates our antidopaminergic hypothesis [15].

In a previous study conducted by Rajkumar et al., CP seed oil has shown anxiolytic properties in normal Wistar rats without causing sedation [28]. The anxiolytic potential of CP has never been evaluated in alcohol withdrawal-induced anxiety. In part 2 of our study, CP decreased alcohol withdrawal-induced anxiety which resulted in the mice spending more time in open arms in EPM and in peripheral squares in the OFT, corroborating the earlier evidence of its anxiolytic property.

Continuous use of alcohol is associated with neuronal loss in the prefrontal cortex which leads to impaired decision-making. The inability of decision-making fosters continuous use of alcohol in addicts despite being aware of problems associated with its continuous use. Koob and Volkow have demonstrated that the neuronal loss in prefrontal cortex observed in alcohol addicts is mediated by glutamate [13]. Antiglutamatergic effect of CP extract was previously demonstrated by Godkar et al. [14]. It can be hypothesised that the antiglutamatergic mechanism of CP contributed to protection against alcohol dependence and withdrawal anxiety observed in our study.

The antianxiety effect of CP could also be attributed to increased brain serotonin levels. In a study conducted by Valecha et al., CP in the doses of 50, 100, 200 mg/kg has been shown to have an anti-depressant effect which was reversed by serotonin synthesis inhibitor para-chlorophenylalanine (p-CPA), indicating that CP increased brain serotonin levels [29].

The neuropharmacological effects of CP seed and seed oil evaluated using in vitro and in vivo studies have been recently reviewed by Bhagya V and Sriranjini J which also substantiate the antioxidant, free radical scavenging and neuroprotective effects of CP [30]. It is possible that antioxidant effect of CP is also contributing to its protective effect against alcohol dependence.

The interpretations of the results observed in this study have limitations as only behavioral paradigms were evaluated. We have not explored its mechanisms of action in the present study. The protective effect of CP in AUD may be mediated by one or more of the antiglutamatergic, antidopaminergic, and antioxidant mechanisms which need exploration. The encouraging results of our study, which is the first of its kind using CP seed oil, provide leads for future research on CP in the area of AUD.

## Conclusions

*Celastrus paniculatus* seed oil at the doses tested in the present study demonstrated a protective effect against alcohol dependence and alcohol withdrawal-induced anxiety in the animal models of AUD. There was a dose-dependent effect and the highest dose of *Celastrus paniculatus* seed oil offered the best protection. The effects in two experimental models were found to be comparable to the standard controls; naltrexone for CPP model and diazepam for alcohol withdrawal anxiety model respectively. The study results provide future directions to explore Celastrus paniculatus in experimental and clinical studies on alcohol use disorder to delineate its potential role in therapy.
